# Proteomic Identification of IPSE/alpha-1 as a Major Hepatotoxin Secreted by *Schistosoma mansoni* Eggs

**DOI:** 10.1371/journal.pntd.0001368

**Published:** 2011-10-25

**Authors:** Maha-Hamadien Abdulla, Kee-Chong Lim, James H. McKerrow, Conor R. Caffrey

**Affiliations:** 1 The Colorectal Research Center, Department of Surgery, King Khalid University Hospital, King Saud University, Riyadh, Saudi Arabia; 2 Sandler Center for Drug Discovery and Department of Pathology, University of California San Francisco, San Francisco, California, United States of America; Institute of Tropical Medicine (NEKKEN), Japan

## Abstract

**Background:**

Eggs deposited in the liver of the mammalian host by the blood fluke parasite, *Schistosoma mansoni*, normally drive a T-helper-2 (Th2)-mediated granulomatous response in immune-competent mice. By contrast, in mice deprived of T-cells and incapable of producing granulomata, egg-secreted proteins (ESP) induce acute hepatic injury and death. Previous work has shown that one such ESP, the T2 ribonuclease known as omega-1, is hepatotoxic *in vivo* in that specific antisera to omega-1 prevent hepatocyte damage.

**Methodology/Principal Findings:**

Using an *in vitro* culture system employing mouse primary hepatocytes and alanine transaminase (ALT) activity as a marker of heptocyte injury, we demonstrated that *S. mansoni* eggs, egg-secreted proteins (ESP), soluble-egg antigen (SEA), and omega-1 are directly hepatotoxic and in a dose-dependent manner. Depletion of omega-1 using a monoclonal antibody abolished the toxicity of pure omega-1 and diminished the toxicity in ESP and SEA by 47 and 33%, respectively. Anion exchange chromatography of ESP yielded one predominant hepatotoxic fraction. Proteomics of that fraction identified the presence of IPSE/alpha-1 (IL-4 inducing principle from *S. mansoni* eggs), a known activator of basophils and inducer of Th2-type responses. Pure recombinant IPSE/alpha-1 also displayed a dose-dependent hepatotoxicity *in vitro*. Monoclonal antibody depletion of IPSE/alpha-1 abolished the latter's toxicity and diminished the total toxicity of ESP and SEA by 32 and 35%, respectively. Combined depletion of omega-1 and IPSE/alpha-1 diminished hepatotoxicity of ESP and SEA by 60 and 58% respectively.

**Conclusions:**

We identified IPSE/alpha-1 as a novel hepatotoxin and conclude that both IPSE/alpha-1 and omega-1 account for the majority of the hepatotoxicity secreted by *S. mansoni* eggs.

## Author Summary

The flatworm disease, schistosomiasis, is a major public health problem in sub-Saharan Africa, South America and East Asia. A hallmark of infection with *Schistosoma mansoni* is the immune response to parasite eggs trapped in the liver and other organs. This response involves an infiltration of cells that surround the parasite egg forming a “granuloma.” In mice deprived of T-cells, this granulomatous response is lacking, and toxic products released by eggs quickly cause liver damage and death. Thus the granulomata protect the host from toxic egg products. Only one hepatotoxic molecule, omega-1, has been described to date. We set out to identify other *S. mansoni* egg hepatotoxins using liver cells grown in culture. We first showed that live eggs, their secretions, and pure omega-1 are toxic. Using a physical separation technique to prepare fractions from whole egg secretions, we identified the presence of IPSE/alpha-1, a protein that is known to strongly influence the immune system. We showed that IPSE/alpha-1 is also hepatotoxic, and that toxicity of both omega-1 and IPSE/alpha-1 can be prevented by first mixing the proteins with specific neutralizing antibodies. Both proteins constitute the majority of hepatotoxicity released by eggs.

## Introduction

Schistosomiasis is a chronic parasitic disease that affects more than 200 million people worldwide [1]. The central pathological characteristic during chronic infection is a granulomatous reaction around trapped parasite eggs in the host's liver, bladder, or intestine [2]. Granulomatous inflammation in the liver may result in fibrosis, scarring, portal hypertension, and in the worst cases hemorrhaging and death [3].

*Schistosoma mansoni* infection in mice is the most common experimental model employed. Approximately five-to-six weeks post-infection, parasite eggs deposited by adult worms induce a T-helper-2 (Th2)-type-polarized immune response [4]. A number of the immunodominant molecules in eggs have been described [5], [6], [7], [8], in addition to the two molecules central to this report (see below). The ability of *S. mansoni* eggs to induce Th2-type differentiation during infection is underscored by the observation that eggs alone, or soluble egg antigen (SEA) released by the eggs through pores in the shell, are sufficient to drive Th2 polarization in naïve uninfected mice [9], [10], [11].

Research from the late 1960s and 1970s has documented that mice lacking T-cells due to genetic loss or surgical removal of the thymus [12], [13], [14], or administration of specific immunosuppressives [15], [16], [17], do not develop a typical granulomatous response to trapped parasite eggs. In these T-cell depleted mice, infection was associated with extensive hepatic parenchymal damage suggestive of a cytotoxic egg product(s) diffusing into hepatic tissue [18]. Histopathology of livers from schistosome-infected immunocompromised mice displayed microvesicular hepatocyte steatosis [18], [19], [20], nuclear degeneration, and hepatocyte apoptosis [21]. Coincident with hepatocyte injury, there is an increase in liver cell transaminases in the plasma [19]. Immunosuppressed mice also have higher mortality once egg deposition in the liver begins [17], [18], [19], [22]. In immunologically intact mice, circumoval granulomata, and antibody responses to released *S. mansoni* egg components, likely protect against hepatocyte damage. Also, hepatotoxicity is prevented in infected T cell-deprived mice by transfer of serum from intact mice immunized with *S. mansoni* eggs or egg homogenate, whilst antisera against other lifecycle stages do not prolong survival [19]. Egg-induced hepatotoxicity appears to be specific to *S. mansoni*; it is not observed during infection of T-cell deprived mice with *S. haematobium* or *S. bovis* [23]. Finally, transfer of sera from *S. japonicum*-infected mice failed to alleviate hepatotoxicity induced by *S. mansoni* eggs in T-cell deprived mice [24].

Research in the 1980s identified several proteins in *S. mansoni* egg antigen preparations based on their electrophoretic mobility and their recognition by sera from mice with chronic infection [18], [25]. Two of these proteins, termed omega-1 and alpha-1, were isolated from *S. mansoni* egg homogenates (SmAE) by cation exchange chromatography in a single salt-eluted fraction that was termed cationic egg fraction 6 (CEF6) [26].

Omega-1 is a 31 kDa monomeric glycoprotein [26] released from *S. mansoni* eggs [27] that has previously been reported to be hepatotoxic [18]. Monospecific antisera against omega-1, which is a highly immunoreactive egg antigen, were protective against hepatocyte damage in T-cell deprived mice [18], [23]. Immunochemical characterization of omega-1 using sera from humans and mice infected with different schistosome species suggested that the antigen is specific to *S. mansoni* [26]. Omega-1 is a functional T2 ribonuclease (RNase) [28]. Cytotoxic RNases (which includes T2 family RNase members) have been found in a wide range of species from bacteria to mammals. A range of biological roles has been suggested, including serving as extra or intracellular cytotoxins and modulating host immune responses [29]. Omega-1 was also reported to drive Th2 polarization in human monocyte-derived dendritic cells (DCs), whereas SEA depleted of omega-1 loses this ability [30]. Omega-1 directly affects both DC morphology and the ability of these antigen-presenting cells to interact physically with CD4 T-lymphocytes [31]. Furthermore, when injected into IL-4 dual reporter mice, omega-1 is a potent inducer of the Th2 response *in vivo* [30].

A second major protein in *S. mansoni* egg homogenates, originally termed alpha-1 [25], was recently reported to be identical to IPSE (IL-4 inducing principle from *S. mansoni* eggs) [32]. IPSE/alpa-1 is a glycoprotein [33] that has been crystallized [34], occurs naturally as a dimer (33–35 kDa), and is enriched in the sub-shell area of *S. mansoni* eggs from where it is secreted into the surrounding tissue [32]. It is not found in the miracidium residing within the egg [35]. Various proteomics analyses have identified IPSE/alpha-1 as an abundant protein in egg secretions [5], [36], [37], [38]. IPSE/alpha-1 binds immunoglobulin and activates naïve basophils, leading to histamine release and facilitating the production of Th2-type cytokines [32]. *In vivo*, IPSE/alpha-1 induces interleukin (IL)-4 secretion from murine basophils in an IgE-dependent but antigen-independent manner [39]. Most recently, IPSE/alpha-1 has been shown to contain a functional C-terminal, monopartite, nuclear localization sequence that binds DNA such that it may alter gene expression in the host cell [40].

We employed a primary hepatocyte *in vitro* culture system to identify and measure direct toxicity of *S. mansoni* eggs and their derived fractions, including pure proteins. Hepatotoxicity of omega-1 was confirmed, and IPSE/alpha-1 was identified as a novel hepatotoxin. Both proteins together account for more than half of the egg-derived toxicity measured.

## Materials and Methods

### Ethics statement

These studies were performed in accordance with the recommendations by the University of California San Francisco Institutional Animal Care and Use Committee. The protocol was approved by the Committee on the Ethics of Animal Experiments of the University of California San Francisco (Permit Number: AN080237-03). All surgery was performed under sodium pentobarbital anesthesia, and all efforts were made to minimize suffering. The protocol followed these guidelines in the study: All U.S. Federal Policy and Guidelines governing the use of laboratory animals, Public Health Service Policy on Humane Care and Use of Laboratory Animals, Guide for the Care and Use of Laboratory Animals, National Academy Press, USDA Animal Welfare Act and Regulations, and NIH Office of Laboratory Animal Welfare Guidelines.

### *In vitro* maintenance of *S. mansoni* and collection of Egg-Secreted Proteins (ESP)

*S. mansoni* eggs were isolated from the livers of female golden hamsters six weeks following infection with 500 cercariae, as previously described [41]. Approximately 0.5 million eggs were washed twice in serum-free RPMI-1640 supplemented with 100 mg/ml streptomycin. Eggs were then resuspended in 2 ml RPMI-1640, and 500 µl aliquots were placed in 12-well culture plates (Costar). Cultures were checked daily by microscopy to ensure sterility. Medium was harvested after 72 h, and centrifuged for 10 min at 200×g to remove eggs. ESP, usually containing approximately 0.5 mg/ml protein by Bradford assay [42], was stored at −80°C. After collection of ESP, egg viability was confirmed by hatching of miracidia; normally >85% of the eggs hatched. Hatching of eggs during the collection period was <1%. SEA was prepared, as described previously [43].

Purified natural omega-1 [30] and recombinant (r)IPSE/alpha-1 proteins [32], and anti-omega-1 (140-3E11) and anti-IPSE/alpha-1 (74-1G2) monoclonal antibodies [32] were kindly supplied by Drs. Gabriele Schramm and Helmut Haas of the Research Center Borstel, Germany. Experiments to deplete ESP and SEA (each 10 µg/ml) of omega-1 and IPSE/alpha-1 involved incubation for 1 h with 5 µg/ml of the respective monoclonal antibodies bound to Protein G Sepharose (GE healthcare Biosciences Pittsburgh, PA). Protein G Sepharose was then removed by centrifugation for 30 min at 100×g.

### Fractionation of ESP by anion exchange chromatography

ESP (2 mg) were added to 2 ml 30 mM Tris-HCl, pH 8.0, and centrifuged at 5000×g for 10 min at 4 C. The supernatant was loaded onto an Hr 5/5 Mono Q column (GE Healthcare), and equilibrated with the same buffer and elute by a 0 to 1 M linear NaCl gradient in six column volumes of Tris-NaCl buffer. Flow-through and eluted fractions (1 ml) were stored at −80°C prior to testing for toxicity with cultured primary hepatocytes (applied volume 100 µl of each fraction to 2×10^5^ hepatocytes/0.5 ml).

### Primary hepatocyte preparation, culture and exposure to egg-derived material

Hepatocytes were isolated from C57/BL6 mice by *in situ* perfusion of liver with collagenase, as described previously [44]. The portal vein was severed to permit outflow followed by cannulation of the inferior vena cava with a 22-guage catheter. The liver was then flushed with a calcium-chelating buffer (liver perfusion medium) for 3 to 5 min, followed by perfusion with collagenase (liver digest medium) for an additional 6–8 min. At the end of the digestion, the liver was removed to a sterile dish and minced thoroughly with a scissors. This crude liver cell isolate was suspended in 25 ml of Dulbecco's modified Eagle's medium/Ham's F-12, filtered through sterile gauze, centrifuged at 70×g for 2 min, and resuspended in Dulbecco's modified Eagle's medium/Ham's F-12. After an additional round of centrifugation and resuspension, hepatocytes were isolated by centrifugation using a 50% Percoll gradient. Hepatocytes were cultured at a density of 2×10^5^/0.5 ml per well in a 12-well culture plate (Costar), previously coated with a 5 mm layer of matrigel (BD Bioscience) [45], which is a tumor biomatrix prepared from the Engelbroth-Holm-Swarm mouse sarcoma [46]. Hepatocytes were allowed to attach for 1 hour at 37°C. Culture plates were gently swirled and the medium containing unattached cells and debris was aspirated. Cultures were then incubated for 72 h in a final volume of 0.5 ml RPMI medium containing 50, 100, or 200 eggs, unfractionated ESP (10 µg/ml) or 100 µl of chromatography fractions. Supernatants were collected and stored at −20°C and analyzed for alanine transaminase (ALT), a serum marker of hepatoxicity [47]) using a Beckman Chemical Analyzer in the Clinical Chemistry Laboratory of the San Francisco Veterans Affairs Medical Center (VAMC).

### Proteomic Analysis of ESP

ESP was fractionated by SDS-PAGE then silver stained [48], [49], and 40 evenly spaced protein bands were sliced out of the gel (Fig S1). The gel slices were then diced into small cubes, reduced and alkylated with dithiothreitol and iodoacetamide, and in-gel digested with trypsin [50], [51]. The resulting peptides were extracted and analyzed by on-line liquid chromatography/mass spectrometry, using an Eksigent nanoflow pump and a Famos autosampler, which were coupled to quadrupole-orthogonal-acceleration-time-of-flight hybrid mass spectrometer (QStar XL or Pulsar, Applied Biosystems). Peptides were fractionated on a reversed-phase column (C18, 0.75×150 mm), and a “5–50% B gradient-in-gradient” was developed in 35 min at a 350-nl/min flow-rate. Solvent A was 0.1% formic acid in water and solvent B was 0.1% formic acid in acetonitrile. Data were acquired in information-dependent acquisition mode: 1 sec MS surveys were followed by 3 sec CID experiments on computer-selected multiply charged precursor ions. Peak lists were generated using Analyst 2.0 software (Applied Biosystems) with the Mascot script 1.6b20 (Matrix Science). Database searches were performed using ProteinProspector v. 5.1.7 (http://prospector2.ucsf.edu) [52]. Searches were performed first on the SwissProt databank (December 16, 2008, 405,506 entries) to evaluate sample purity, followed by searching in the *S. mansoni* database SchistoDB v. 4.0 (www.schistodb.net; 13,174 entries downloaded July 2009). Batch-Tag settings were selected for samples prepared with trypsin allowing a maximum of one missed cleavage and no non-specific cleavages. Peptide modifications searched for included carbamidomethyl (Cys) as the only fixed modification, and up to two variable modifications from among the following: oxidation (Met), acetyl (N-term), oxidized acetyl (N-term), pyroglutamate (Gln) and Met-loss (N-term). The mass accuracy considered was 200 ppm, and 300 ppm for the precursor and fragment ions, respectively. The following acceptance scores for database matches were required: a minimum protein score of 22, a minimum peptide score of 15, and a maximum expectation value of 0.02 required for both peptide and protein identification. These criteria resulted in an approximate 2% false determination rate. Protein identifications are reported with a minimum of two peptide matches per protein. For the analysis of ESP anion exchange fraction #11, the maximum expectation value was changed to 0.05, and no decoy proteins were identified using these acceptance criteria.

## Results

### *S. mansoni* eggs and their secretions are hepatotoxic *in vitro*

**Figure 1:**
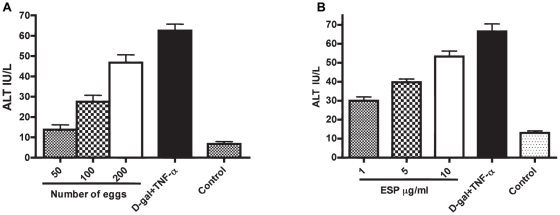
*S. mansoni* eggs and their secretions induce dose-dependent hepatotoxicity *in vitro*. Mouse primary hepatocytes cultured in 0.5 ml Dulbecco's modified Eagle's medium were co-incubated with different numbers of (A) eggs or (B) ESP. After 72 h, alanine tranaminase (ALT), a biomarker for hepatotoxicity, was measured. A combination of 5 mM D-galactosamine hydrochloride (D-gal) and 1 µg/ml rTNF-α (D-gal/TNF-α) was used as a known hepatotoxic control. As a negative control, hepatocytes were incubated with PBS. Data are displayed as the mean ± S.D. from two experiments each performed in duplicate.

To measure hepatotoxicity caused by parasite eggs ESP, and their chromatographic fractions, we employed an *in vitro* system involving murine primary hepatocytes cultured on matrigel. ALT was employed as a hepatoxicity biomarker. With parasite eggs, measurements were taken 24, 48 and 72 h. No alteration in ALT levels was seen at 24 or 48 h (not shown); however, by 72 h, ALT had increased markedly and in a dose-dependent manner (Figure 1A). Dose-dependent hepatocellular injury elicited by ESP was also measured after 72 h (Figure 1B).

**Figure 2:**
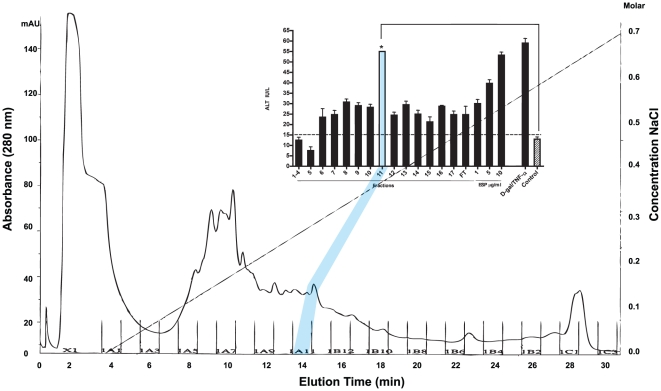
Anion exchange chromatography of ESP identifies a major hepatotoxic fraction. ESP was fractionated by Mono Q anion exchange chromatography. The fraction not bound to the column (flow-through (‘FT’)) and each subsequent eluted fraction were incubated with mouse primary hepatocytes in 0.5 ml Dulbecco's modified Eagle's medium. After 72 h, ALT, a biomarker for hepatotoxicity, was measured. Fraction #11 induced significant liver cell injury *P<0.05 compared to negative control cultures employing PBS. Other fractions (6–10, 12–17) had some hepatotoxic effect. No hepatotoxic effect was present in fractions 1–5. Total ESP (10 µg/ml) and D-gal/TNF-α (5 mM/1 ug/ml) were included as positive controls. Data are displayed as the mean ± S.D. from two experiments each performed in duplicate.

To aid identification of those ESP constituents responsible for hepatotoxicity, ESP was fractionated by Mono Q anion exchange chromatography. The flow-through (component not bound to the column), and each of the eluted fractions (100 µL), was co-incubated with cultured hepatocytes. At 72 h, fraction #11 induced an approximate two-fold greater release of ALT relative to the other eluted fractions and flow-through, such that it was the equivalent of 10 µg/ml unfractionated ESP (Figure 2).

### Proteomic analysis of ESP and hepatotoxic fraction #11: identification of IPSE/alpha-1

SDS-PAGE and tryptic digestion followed by mass spectrometry of ESP and hepatotoxic fraction #11 identified 99 and nine proteins, respectively (Tables 1 and 2). Previously, total proteomic analysis of ESP identified 188 proteins [36], and many are common between this and the present dataset (Table 1). Among the nine proteins identified in fraction #11 were metabolic enzymes involved in glucose metabolism, glycogen storage, in addition to chaperones. IPSE/alpha-1 was also identified; and, given its potent immunomodulatory properties [32], [34], was of immediate interest in discovering of whether or not it was hepatotoxic.

**Table 1:** Proteins identified in ESP by geLC-MS/MS and searching in SchistoDB.

Protein Name	Accession #	Peptides	% Cov^*^	Score	Expect	MW (kDa)	Gel Band	
phosphoenolpyruvate carboxykinase	Smp_005880	34	46	4.76	2.50E-08	70.4	8, 13, 14, 19, 20, 30	++
macroglobulin/complement	Smp_089670	30	12	4.57	7.50E-08	222.2	34–36	−
enolase (2-phosphoglycerate dehydratase)	Smp_024110	22	42	4.96	1.40E-08	47.0	8, 9, 12, 24, 25	++
fructose 1,6 bisphosphate aldolase	Smp_042160.2	20	53	4.75	3.40E-08	39.6	20–22	++
beta tubulin	Smp_035760	17	37	5.9	2.60E-10	49.8	12–14, 19, 20	++
glutathione S-transferase 26 kDa (GST 26) (GST class-mu)	Smp_102070	20	68	4.62	6.00E-08	25.3	15–18	++
phosphoglycerate mutase	Smp_096760	17	56	5.19	5.30E-09	28.4	18	++
thioredoxin peroxidase (Prx 1)	Smp_059480	17	51	5.11	7.60E-09	21.1	12–14, 20, 21	++
glutathione S-transferase 28 kDa (GST 28) (GST class-mu)	Smp_054160	19	57	4.46	1.20E-07	23.8	16–18	++
malate dehydrogenase	Smp_035270.2	18	34	5.98	1.90E-10	36.2	10, 11, 14–16, 20, 21	++
triosephosphate isomerase	Smp_003990	16	43	4.81	2.70E-08	28.1	16–18	+
purine nucleoside phosphorylase	Smp_090520	15	31	4.76	3.30E-08	45.2	6, 14, 19	++
family C56 non-peptidase homologue (C56 family)	Smp_082030	10	69	6.02	1.50E-10	19.1	12, 13	++
alpha tubulin	Smp_090120.1	9	24	5.52	1.30E-09	50.0	9, 12–15, 26	++
alpha-galactosidase/alpha-n-acetylgalactosaminidase	Smp_179260	12	10	3.57	1.10E-06	108.5	19, 20, 25, 26	+
**interleukin-4-inducing protein precursor (IPSE/alpha-1)**	Smp_112110	9	49	4.83	2.50E-08	15.4	13, 14, 19–21, 24	++
**hepatotoxic ribonuclease omega-1 precursor**	Smp_193860	13	63	3.31	1.60E-06	15.1	12, 18, 19	++
aminopeptidase PILS (M01 family)	Smp_174530	14	13	2.9	1.40E-05	110.8	29, 31, 34, 35	−
heat shock protein 70	Smp_106930.1	11	19	4.72	1.50E-08	69.0	10, 16, 17, 24, 32, 39	++
thioredoxin glutathione reductase	Smp_048430	9	16	4.3	2.40E-07	64.8	8, 27, 28–31	++
peroxiredoxin, Prx-2	Smp_158110	12	30	3.71	3.60E-07	21.7	8, 12, 13	++
peroxiredoxins, prx-1, prx-2, prx-3	Smp_062900	5	14	4.5	1.00E-07	21.7	12–14	−
proteasome subunit alpha 2 (T01 family)	Smp_067890	10	45	3.53	4.40E-06	25.8	16	−
ATP:guanidino kinase (Smc74), putative	Smp_194770	12	16	4.06	2.20E-07	94.9	19–22, 32, 33	+
annexin	Smp_164100	12	28	3.36	4.00E-06	40.4	13–15, 22	+
expressed protein	Smp_160560	9	13	3.29	1.70E-05	79.5	19, 20, 36	−
malate dehydrogenase	Smp_047370	10	26	3.42	6.10E-07	36.3	8, 17, 19, 20	++
thimet oligopeptidase (M03 family)	Smp_029500	8	12	4.34	1.00E-07	77.7	9, 18, 20, 24, 25, 30	−
actin	Smp_046590	7	17	4.87	1.70E-08	41.7	8, 10, 11, 13, 19	++
serpin	Smp_090080	13	25	2.27	2.10E-05	46.0	20, 25–30	+
ferritin light chain	Smp_087760	8	25	3.43	5.90E-07	20.2	11, 14	−
nucleoside diphosphate kinase	Smp_092750	11	52	3.22	2.60E-07	16.9	11–13	+
phosphoglycerate kinase	Smp_187370	7	33	5.8	3.90E-10	18.5	11, 12, 19, 20, 25	++
expressed protein	Smp_150240	10	18	3.21	5.10E-07	31.1	25, 31, 32, 34	−
glutathione-s-transferase omega	Smp_152710.2	8	34	2.9	1.40E-05	27.0	17, 18	−
expressed protein	Smp_096790	6	20	4.72	3.90E-08	22.0	9, 13, 14, 16	−
histone H2B	Smp_036220	5	42	3.77	2.20E-06	13.6	9, 11, 12	+
aldo-keto reductase	Smp_053220.1	7	18	5.07	3.60E-09	35.5	9, 20	+
anti-inflammatory protein 16	Smp_113760	10	60	3.04	2.80E-06	9.0	9–13	−
expressed protein	Smp_138760	5	25	4.81	1.80E-08	32.3	14, 19, 20	+
pyruvate kinase	Smp_065610.1	7	16	3.23	5.00E-07	54.8	14, 20	+
filamin	Smp_000100	3	2	5.26	3.90E-09	261.0	25, 27	−
expressed protein	Smp_179630	4	20	4.08	5.90E-07	19.5	18, 25, 26	−
histone H4	Smp_053290	8	56	2.49	4.50E-06	11.4	9–11	++
heat shock protein (Major egg antigen, P40)	Smp_049250	7	21	2.38	1.30E-04	40.2	14, 22	++
superoxide dismutase [Cu-Zn]	Smp_176200	3	44	5.32	3.10E-09	15.9	11	++
dihydrolipoamide dehydrogenase	Smp_046740	6	14	2.49	2.80E-05	52.9	10, 11, 25, 26	++
proteasome subunit alpha 6 (T01 family)	Smp_130110	4	5	3.64	3.90E-06	76.9	10, 17	−
thioredoxin	Smp_008070	4	40	4.05	2.50E-07	11.9	6–8	−
expressed protein	Smp_011030	3	16	4.32	2.10E-07	20.1	6, 7, 14	++
expressed protein	Smp_088720	5	20	3.09	8.40E-07	27.4	16, 18, 19	−
transaldolase	Smp_070600	7	18	1.92	4.70E-05	37.8	18–20, 22	+
calcium-binding protein	Smp_096390	3	21	4.48	1.10E-07	16.7	9, 10	−
calpain (C02 family)	Smp_157500	3	2	4.3	1.00E-07	172.9	12, 13	++
22.6kDa tegument-associated antigen	Smp_086470	3	12	3.77	2.00E-08	21.6	4, 8, 9	++
phosphomannomutase	Smp_087860	3	14	4.01	8.10E-07	28.1	16, 18	+
glucose-6-phosphate isomerase	Smp_022400	3	6	3.37	2.10E-07	61.1	26	+
14-3-3 protein, putative	Smp_009760	8	30	2.06	1.20E-05	28.4	18	++
200-kDa GPI-anchored surface glycoprotein	Smp_017730	3	2	3.6	1.70E-06	186.5	34	−
uridine phosphorylase	Smp_082420	2	12	5.15	6.20E-09	32.8	10, 18	−
cyclophilin	Smp_040130	5	22	3.54	1.90E-07	17.7	8, 11–13	++
ferritin light chain	Smp_047650	5	16	3.1	6.90E-07	19.7	36	+
alpha-glucosidase	Smp_133210	7	8	1.72	3.10E-05	102.5	32–34	−
expressed protein	Smp_187410	2	8	5.42	1.00E-10	32.0	30, 31	−
glyceraldehyde-3-phosphate dehydrogenase (GAPDH)	Smp_056970.1	4	12	2.78	3.10E-06	36.4	11, 20, 21	++
glycogen phosphorylase	Smp_143840	8	11	1.75	1.50E-04	80.0	8, 9, 31, 33	++
proteasome subunit alpha 3 (T01 family)	Smp_092280	3	13	3.46	7.20E-08	28.1	17, 18	−
ribose-5-phosphate isomerase, putative	Smp_148770	3	3	2.9	4.90E-06	159.4	18	−
venom allergen-like (VAL) 5 protein	Smp_120670	2	11	3.87	8.70E-07	16.4	16	−
expressed protein	Smp_170410	6	19	2.19	2.90E-06	29.3	25, 26, 29	−
leucine aminopeptidase (M17 family)	Smp_030000	6	11	2.28	1.30E-05	56.7	26–28	−
methylthioadenosine phosphorylase	Smp_028190	3	11	3.55	1.50E-07	34.6	12, 13	−
superoxide dismutase [Mn]	Smp_056440	3	11	2.68	1.20E-05	24.3	15	++
L-lactate dehydrogenase, putative	Smp_038950	3	9	3.62	4.20E-07	36.0	8, 14	+
inorganic pyrophosphatase, putative	Smp_135950	4	3	2.05	3.40E-05	180.7	20	+
alkaline phosphatase	Smp_155890	4	12	2.43	2.00E-06	47.1	28, 30, 31	−
phosphoglucomutase	Smp_180530.3	2	5	3.78	4.10E-07	62.4	28, 29	−
xylosyltransferase	Smp_128310.1	2	3	4.01	9.10E-08	89.6	6, 10	−
proteasome subunit beta 2 (T01 family)	Smp_074500.2	2	11	3.23	1.80E-06	22.8	14	−
aldehyde dehydrogenase	Smp_050390	4	9	1.81	3.00E-04	53.8	8, 19, 26	++
NAD dependent epimerase/dehydratase	Smp_089370	4	16	2.19	7.10E-06	29.8	5, 9, 10	−
PwLAP aminopeptidase (M17 family)	Smp_083870	7	13	1.49	2.90E-05	59.7	27, 28	−
annexin	Smp_045560	5	18	2.07	2.30E-05	36.9	19, 20	++
SmVAL26	Smp_154260	2	15	2.52	1.40E-04	20.5	10, 11	−
proteasome catalytic subunit 2 (T01 family)	Smp_073410	2	7	2.69	8.00E-06	30.5	14, 15	−
ribosomal protein related	Smp_154690	2	4	2.22	6.50E-05	42.6	26	−
dynein light chain	Smp_056780	2	27	2.81	1.40E-06	10.8	8, 9	−
cytochrome c	Smp_033400	2	18	2.99	1.80E-06	12.0	9	−
SmVAL27	Smp_154290	3	20	1.92	3.10E-05	20.7	12	−
calmodulin	Smp_026560.1	1	10	3.75	1.30E-06	17.2	12	+
titin, putative	Smp_126240	3	1	1.47	4.20E-05	671.4	35, 36	−
peptidylglycine mono-oxygenase	Smp_145300	3	7	1.46	2.10E-04	26.5	25, 29	++
SmVAL23	Smp_160250	1	6	3.36	6.60E-06	22.8	12	−
inosine triphosphate pyrophosphatase	Smp_063120.1	2	10	1.77	6.40E-05	21.2	13	−
Sodium/potassium-transporting ATPase subunit beta	Smp_033550	2	9	1.73	1.40E-05	32.1	20	−
proteasome subunit alpha 7 (T01 family)	Smp_076230	2	13	2.33	1.20E-05	29.7	18, 19	−
low-density lipoprotein receptor (LDL)	Smp_159420	4	4	1.17	3.50E-04	102.7	35	−
arginase	Smp_059980	2	7	2.45	1.40E-06	39.9	11, 20	+
heme binding protein	Smp_016730	3	22	1.55	2.10E-05	20.6	14	+
dipeptidyl-peptidase III (M49 family)	Smp_019010	2	3	1.81	7.90E-05	76.2	31, 32	+
expressed protein	Smp_153230	2	2	1.71	9.90E-06	146.3	36	−
ATP synthase alpha subunit mitochondrial	Smp_002880.1	4	9	1.55	1.70E-05	59.6	26	−
adenylate kinase	Smp_071390	2	10	1.52	1.30E-04	22.3	14	+

Note: cutoff above 70% (++), below (+) and (−) not found.

**Table 2:** Proteins identified in hepatotoxic fraction #11 by LC/MS/MS analysis after SDS-PAGE fractionation and in-gel digestion, or after in-solution digestion.

Gel-band	Rank	Protein Name	Acc #	Num Unique	% Cov	Best Disc Score	Best Expect Val	Protein MW
1	[1]	glycogen phosphorylase	Smp_143840	32	50.7	5.36	2.60E-09	80035
1	[2]	glycogen phosphorylase	Smp_143850	7	51.1	4.32	2.10E-07	16835
2	[1]	heat shock protein 70	Smp_182190.2	31	41.3	5.61	8.90E-10	69830
3	[1]	aldehyde dehydrogenase	Smp_050390	5	11.6	4.33	2.10E-07	53763
3	[2]	expressed protein	Smp_170410	2	4.2	2.7	1.70E-05	29253
4	[1]	heat shock protein (Major egg antigen (P40)	Smp_049250	10	25.1	4.76	3.30E-08	40192
5	[1]	heat shock protein 70	Smp_182190.2	39	52	5.48	1.50E-09	69830
6	[1]	aldehyde dehydrogenase	Smp_050390	11	28.9	4.9	1.90E-08	53763
6	[2]	utp-glucose-1-phosphate uridylyltransferase 2	Smp_133600	4	12.8	4.92	1.70E-08	52729
6	[3]	expressed protein	Smp_170410	3	9.2	3.57	5.30E-06	29253
6	[4]	heat shock protein 70	Smp_106930	2	3.3	0.99	2.30E-04	69004
7	[1]	heat shock protein (Major egg antigen (P40)	Smp_049250	2	6.9	2.86	1.10E-04	40192
8	[1]	heat shock protein (Major egg antigen (P40)	Smp_049250	4	10.8	2.99	6.20E-05	40192
8	[2]	fructose-1,6-bisphosphatase-related	Smp_097370	3	6.1	0.64	0.01	37472
9	[1]	**interleukin-4-inducing protein precursor (IPSE/ALPHA-1)**	Smp_112110	3	25.4	4.82	2.60E-08	15358
9	[2]	heat shock protein	Smp_049250	2	4.7	2.75	1.70E-04	40192
in solution	[1]	heat shock protein 70	Smp_106930	21	33.2	5.7	5.90E-10	69004
in solution	[2]	heat shock protein, (Major egg antigen (P40)	Smp_049250	8	17.7	4.15	4.50E-07	40192
in solution	[3]	glycogen phosphorylase	Smp_143840	5	6.6	3.12	3.50E-05	80035
in solution	[4]	aldehyde dehydrogenase	Smp_050390	5	11.6	2.22	4.30E-05	53763
in solution	[6]	macroglobulin/complement	Smp_089670	2	0.9	0.72	0.0054	222171

### *In vitro* confirmation that Omega-1 is hepatotoxic

**Figure 3:**
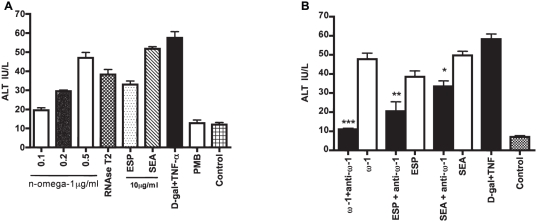
Omega (ω)-1 exhibits dose-dependent hepatotoxicity that is neutralized by a specific monoclonal antibody. (A) Hepatocyte cultures (0.5 ml in Dulbecco's modified Eagle's medium were co-incubated with various amounts of purified native omega-1, and egg-derived material (10 µg/ml). After 72 h, ALT, a biomarker for hepatotoxicity, was measured. Polymyxin B (80 µg/ml) was included in co-incubations with omega-1 to neutralize any potential LPS. Control cultures contained polymyxin B alone. *Aspergillus oryzae* T2 RNAse (25 U/ml) was also used as a comparison in light of omega-1's described T2 RNase activity. A combination of 5 mM D-galactosamine hydrochloride (D-gal) and 1 µg/ml rTNF-α (D-gal/TNF-α) was employed as a known hepatotoxic control and negative control cultures used PBS. (B) Pre-incubation of a specific monoclonal antibody (5 µg/ml) with omega-1 abolished the latter's toxicity and respectively decreased cytotoxicity of ESP and SEA by 47 and 33%. Data are presented as the means ± SD from two independent experiments each performed in duplicate. *P<0.02, **P<0.001 and ***P<0.0001 using a one-sided paired Student's t-test.

Omega-1 is an egg-secreted glycoprotein with RNase activity and *in vivo*-demonstrated hepatotoxicity [18], [28]. To measure direct toxicity to primary cultured hepatocytes, purified native omega-1 was co-incubated with primary hepatocytes. At 72 h, a dose-dependent release of ALT was measured (Figure 3A). Likewise, *Aspergillus oryzae* T2 RNase (25 U/µl) (Invitrogen, # 18031-013 Carlsbad, CA) was toxic. Importantly, pre-incubation of pure omega-1 with a monoclonal anti-omega-1 antibody bound to Protein G Sepharose abolished cytotoxicity (Figure 3B). Depleting ESP and SEA with the same antibody decreased toxicity by 47 and 33%, respectively. All reductions in hepatoxicity were statistically significant (Figure 3B).

### IPSE/alpha-1 is also a hepatotoxin *in vitro*

**Figure 4:**
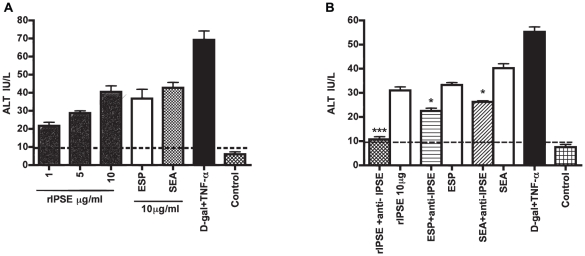
IPSE exhibits dose-dependent heptotoxicity that is neutralized by specific monoclonal antibody. (A) Hepatocytes were co-incubated with various amounts of rIPSE. After 72 h, the presence of ALT in the medium was measured. As positive controls, cells were co-incubated with 10 µg/ml each of ESP or SEA. A combination of 5 mM D-galactosamine hydrochloride (D-gal) and 1 µg/ml rTNF-α (D-gal/TNF-α) was employed as a known hepatotoxic control and negative control cultures used PBS. (B) Pre-incubation of a specific monoclonal antibody (5 µg/ml) with rIPSE abolished the latter's toxicity and respectively decreased cytotoxicity of ESP and SEA by 32 and 35%. Data are displayed as the mean ± S.D. from two experiments each performed in duplicate. *P<0.05, **P<0.001 and ***P<0.0001 using a one-sided paired Student's t-test. The hatched line represents the control baseline.

IPSE/alpha-1 was an abundant protein in the hepatotoxic fraction #11 from anion exchange chromatography (Table 2). Recombinant IPSE/alpha-1 was added to hepatocyte cultures, and a dose-dependent toxicity was measured at 72 h ALT levels that was significantly elevated relative to negative controls (Figure 4A). Similar to that found for omega-1, specific neutralization of rIPSE/alpha-1 with an anti-rIPSE/alpha-1 monoclonal antibody abolished activity and decreased the cytotoxicity of ESP and SEA by 32 and 35%, respectively (Figure 4B). All reductions in hepatoxicity were statistically significant.

### Omega-1 and IPSE/alpha-1 are major hepatotoxins in ESP and SEA

**Figure 5:**
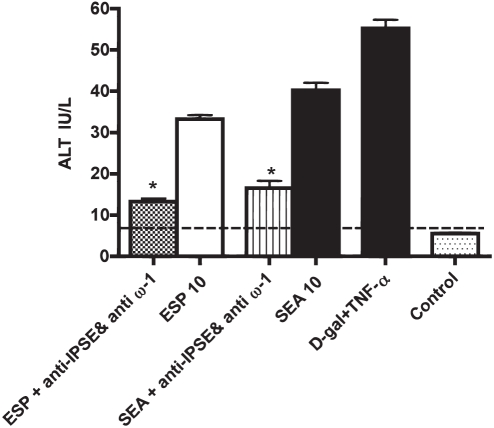
Omega-1 and IPSE are major hepatotoxins in ESP and SEA. Hepatocytes were co-incubated with 10 µg/ml ESP or SEA, or with ESP or SEA first depleted of both omega-1 and IPSE with a mixture of anti-IPSE and anti-omega-1 monoclonal antibodies (each 5 µg/ml). After 72 h, the presence of ALT in the medium was measured. A combination of 5 mM D-galactosamine hydrochloride (D-gal) and 1 µg/ml rTNF-α (D-gal/TNF-α) was employed as a known hepatotoxic control and negative control cultures used PBS. Depletion of both IPSE and omega-1 diminished the toxicity of ESP and SEA by 60% and 58%, respectively. Data are displayed as the mean ± S.D. from two experiments each in duplicate. * P<0.05 for values significantly different from ESP and SEA is based on paired analysis (one sided paired Student's t-test).

To measure the combined contributions of omega-1 and IPSE/alpha-1 to the hepatotoxicity of ESP and SEA *in vitro*, both egg-derived preparations were depleted of both omega-1 and IPSE/alpha-1 with specific monoclonal antibodies prior to incubation with hepatocytes. The combination of both antibodies diminished hepatotoxicity of ESP and SEA by 60 and 58%, respectively (Figure 5).

## Discussion

The pathogenesis of hepatic schistosomiasis is due to the host's granulomatous response to eggs deposited in the liver [2]. The initial cellular granuloma is characterized by the presence of activated macrophages, lymphocytes, and eosinophils, as reviewed in both Agnew and Pearce [23], [53]. Over time, granulomata become fibrotic, and their accumulation in periportal areas, as is the case in chronic *S.mansoni* infection, can lead to portal hypertension, hemorrhaging, and death [3]. Ironically, in the absence of a granulomatous response, experimental hepatic schistosomiasis in mice leads to a more acute and lethal disease [17], [18], [19], [20], [22], [54]. The understanding from such observations is that schistosome eggs release hepatotoxins, toxins that are normally prevented from diffusing by circumoval granulomata. To date, the only hepatotoxin characterized in *S. mansoni* eggs is omega-1 [18] which is RNaseT2 [28], that also induces a Th2 response [30].

We established an *in vitro* primary hepatocyte culture system using ALT as the metric for cell injury to identify egg components with direct hepatotoxicity. We first confirmed the toxicity of *S. mansoni* eggs and their derivatives, ESP and SEA, and then showed that pure native omega-1 is hepatotoxic *in vitro*, consistent with previous *in vivo* observations. Based on the present system, omega-1 is a major toxin released by *S. mansoni* eggs, as depletion of ESP or SEA with a specific monoclonal antibody decreased ALT levels by 47 and 33%, respectively.

To search for additional hepatotoxins, we combined anion exchange chromatography of ESP with proteomics. A single hepatotoxic fraction (#11) was identified which contained a short list of nine proteins. IPSE/alpha-1 stood out as a molecule of interest given its potent immunomodulatory properties [39], [55]. Subsequent characterization of pure rIPSE/alpha-1 demonstrated that the molecule is indeed directly hepatotoxic. The finding was confirmed using a specific monoclonal antibody that essentially neutralized IPSE/alpha-1 toxicity while decreasing the cell injury produced by both ESP and SEA by approximately one-third. Further depletion of ESP and SEA with a combination of monoclonal antibodies targeting both omega-1 and IPSE/alpha-1 indicated that approximately 60% of the toxicity of the egg-derived material is due to these two proteins. This leaves room for additional hepatotoxins to be identified, perhaps by different chemical and physical separation approaches. We also note that although both omega-1 and IPSE/alpha-1 were identified in the total ESP proteome, only IPSE/alpha-1 was subsequently found in the single hepatotoxic fraction #11. This suggests that omega-1 was below the mass spectrometry detection limits used to identify proteins.

Recently, IPSE/alpha-1 was reported to be internalized by Chinese hamster ovary cells (CHO) and primary monocyte-derived dendritic cells, but not by peripheral blood basophils [40]; and in each case without apparent toxicity. This suggests that host cell-specific factors determine how cells interact with and respond to IPSE/alpha-1. Such factors might explain why IPSE/alpha-1 is directly toxic to hepatocytes. Studies to understand the mechanism of hepatoxicity induced by IPSE/alpha-1, and other hepatotoxins such as omega-1, can now be undertaken with the present *in vitro* system. Ribonuclease activity is often associated with cytotoxicity, and Steinfelder et al noted that omega-1 was initially characterized as a hepatotoxic agent from *S. mansoni* [28]. Nevertheless, the Th2-promoting activity of omega-1 cannot be explained by a cytotoxic effect, as the molecule failed to induce a detectable reduction in dendritic cell viability. The exact mechanism(s) by which the ribonuclease activity of omega-1 may promote Th2 responses is currently under investigation [31].

The results presented here underscore the paradox of the granulomatous response in hepatic schistosomiasis. Though detrimental to the host in the longer term due to its contribution to disease sequelae such as portal hypertension, it nevertheless protects against more acute hepatocyte injury resulting from toxins released by the schistosome egg.

## Supporting Information

Figure S1 **SDS-PAGE preparation of ESP.** ESP (20 µg) was loaded into SDS-PAGE, the gel was silver stained, then sliced into 40 bands as indicated for in gel trypsin digestion and peptide sequencing by LC-MS/MS. The picture shows Fluorescence image for SyproRuby stained gel.
(DOC)
