# Supplementary material for: Proteomic Identification of IPSE/alpha-1 as a Major Hepatotoxin Secreted by Schistosoma mansoni Eggs
Source: PLoS Negl Trop Dis. 2011 Oct 25;5(10):e1368. doi: 10.1371/journal.pntd.0001368 (PMC3201919; doi:10.1371/journal.pntd.0001368)
Supplement: Figure S1 — SDS-PAGE preparation of ESP. ESP (20 µg) was loaded into SDS-PAGE, the gel was silver stained, then sliced into 40 bands as indicated for in gel trypsin digestion and peptide sequencing by LC-MS/MS. The picture shows Fluorescence image for SyproRuby stained gel. (DOC) [file pntd.0001368.s001.doc]

**Supplementary Fig-1**

**
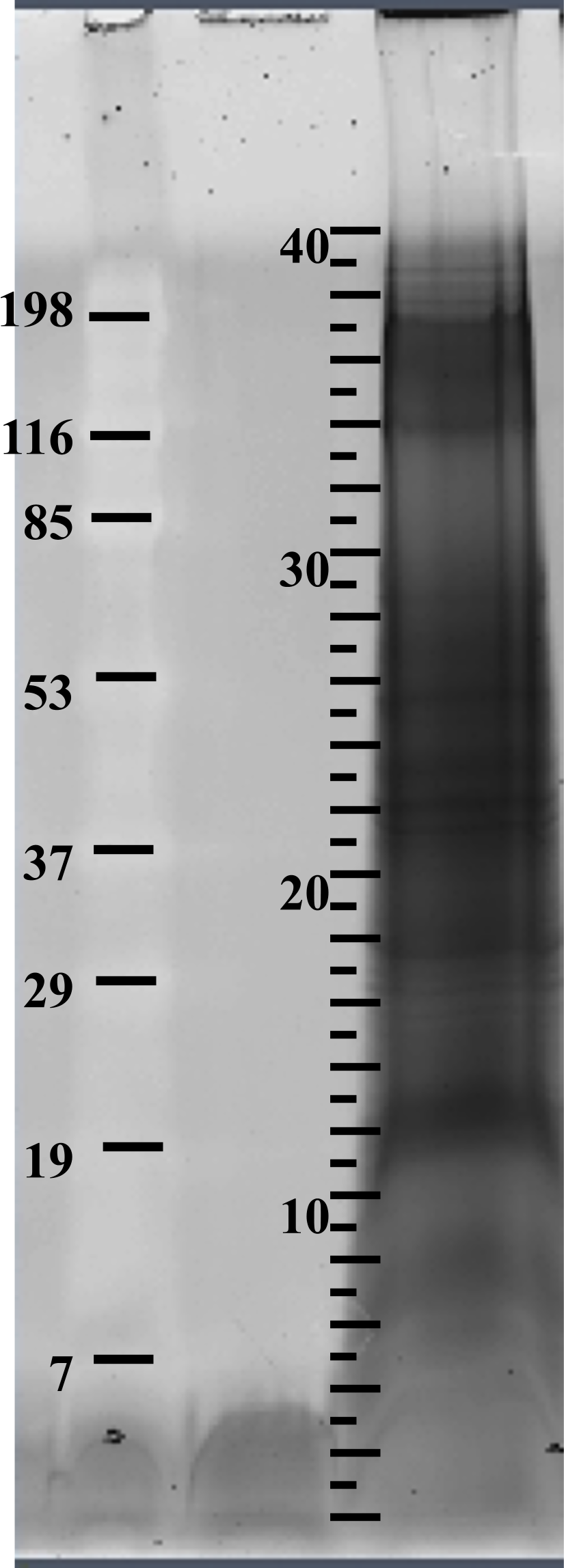
**

**Fig-1. SDS-PAGE preparation of ESP.** Fluorescence image for SyproRuby stained gel, containing 20 µg total protein. The gel was silver stained, then sliced into 40 bands as indicated for in gel trypsin digestion and peptide sequencing by LC-MS/MS.
